# Investigation on Blind Tip Reconstruction Errors Caused by Sample Features

**DOI:** 10.3390/s141223159

**Published:** 2014-12-05

**Authors:** Jiahuan Wan, Linyan Xu, Sen Wu, Xiaodong Hu

**Affiliations:** State Key Lab of Precision Measuring Technology and Instruments, Tianjin University, Tianjin 300072, China; E-Mails: wanjiahuan@tju.edu.cn (J.W.); senwu@tju.edu.cn (S.W.); xdhu@tju.edu.cn (X.H.)

**Keywords:** blind tip reconstruction, 3D tip shape, AFM, tip characterizer

## Abstract

Precision measurements of a nanoscale sample surface using an atomic force microscope (AFM) require a precise quantitative knowledge of the 3D tip shape. Blind tip reconstruction (BTR), established by Villarrubia, gives an outer bound with larger errors if the tip characterizer is not appropriate. In order to explore the errors of BTR, a series of simulation experiments based on a conical model were carried out. The results show that, to reconstruct the tip precisely, the cone angle of the tip characterizer must be smaller than that of the tip. Furthermore, the errors decrease as a function of the tip cone angle and increase linearly with the sample radius of curvature, irrespective of the tip radius of curvature. In particular, for sharp (20 nm radius) and blunt (80 nm radius) tips, the radius of curvature of the tip characterizer must be smaller than 5 nm. Based on these simulation results, a local error model of BTR was established. The maximum deviation between the errors derived from the model and the simulated experiments is 1.22 nm. Compared with the lateral resolution used in the above simulated experiments (4 nm/pixel), it is valid to ignore the deviations and consider the local error model of BTR is indeed in quantitative agreement with the simulation results. Finally, two simulated ideal structures are proposed here, together with their corresponding real samples. The simulation results show they are suitable for BTR.

## 1. Introduction

Atomic force microscope (AFM) is the most prevalent technique for studying the surface properties of materials from the micron all the way down to the atomic level in a variety of science and technology areas. It can be used to study insulators [1], as well as semiconductors [2] and conductors [3]. Unfortunately, some limitations caused by the geometry of the scanning tip degrade the accuracy of the AFM images which are distorted by the dilation effect between the tip and the actual sample surface [4–6]. Since the touching segments of the tip are not always the vertex during the scanning process, the deflection of the cantilever is not only affected by the actual sample surface but also affected by the tip shape. Furthermore, such image distortions are increased as soon as the dimensions of the AFM tip becomes comparable to the size of the sample features. The distorted image will have a great impact on roughness analysis [7], dimensional measurements [8], hardness measurements [9], *etc*. That is why the 3D tip shape needs to be known precisely in advance in order to obtain an accurate representation of the sample surface [10,11]. In addition, the tip shape also effects the measurements that determine mechanical properties of surfaces and forces, such as Young's modulus and adhesion [12,13]. Aside from tip shape, calibrations for cantilever deflection [14,15] and the spring constant of the AFM cantilever [16–18] are also crucial to the measurements of mechanical properties of surfaces and forces. These types of calibrations are very important for the quantitative measurements in AFM.

Until recently, there have been many published papers devoted to solve the above problem which broadly fall into two groups, directly imaging the tip without AFM and indirectly calibrating the tip while using AFM. Among the two groups, various mathematical methods are used together. Direct microprobe imaging methods mostly involve either a high-resolution scanning electron microscope (SEM) [19] or a transmission electron microscope (TEM) [20]. These methods are limited to the 2D profile imaging. In addition, they may show other disadvantages such as SEM probe-specimen “convolution” effects, sample contamination induced by electron beam [21], and unstable resolution caused by material charging during measuring process. The methods of indirect imaging of the tip could be mainly divided into two groups based on the choice of the tip characterizer. The first group uses the well-calibrated tip characterizer, such as the sharp-edged calibration structures [22] and polystyrene nanospheres [23–25]. This method relies completely on the accuracy of the calibrated geometry of the tip characterizer which is significantly restricted by the nanometer scale measurement ability of the instrument. In addition, the tip characterizer may suffer from uncertain wear or contamination during the scanning process which makes the previous calibration unreliable. Another group uses tip characterizer without calibration. This method can extract tip shape from an AFM image by various mathematical algorithms without calibration of the tip characterizer, such as the deconvolution algorithm [26], expectation maximization (EM) algorithm [27] and morphological estimation algorithm [28].

Among the above methods, the most promising one is the Blind Tip Reconstruction (BTR) algorithm which is able to determine the best upper bound of the tip shape using mathematical morphology operations. With suitable tip characterizer, this method can not only be used to reconstruct standard silicon or silicon nitride AFM tips, but also be used to reconstruct carbon nanotube AFM tips [29,30] which tend to have smaller diameters and much higher aspect ratios than standard AFM tips. Here, we concentrate on the BTR algorithms proposed by Villarrubia [31,32] and verified by Dongmo [33]. It is most widely studied [34,35] and further regularized to reconstruct the flared tip by using a dexel representation [36,37]. This algorithm can reconstruct an upper bound of the tip shape from an AFM image obtained by scanning any samples. However, with the upper bound, the reconstruction image will be below the actual sample surface which is far from our expectation and certainly not be beneficial to further analysis like the calculation of organic molecule models in biology, the determination of surface roughness and grain size in material science, the identifications of critical dimension (CD) in semiconductor industry. Therefore, in order to determine the real sample surface geometry, it is better to obtain a more accurate 3D tip shape, *i.e.*, the minimum upper bound by using the suitable tip characterizer.

However, among all the published papers about BTR, there are only some fuzzy requirements of the tip characterizer since the BTR method works without prior knowledge of the sample. This leads the algorithm to work excellent in theory but not always in practice. For example, the simulated experiment illustrated by Villarubia in 1997 [32] was done on a granular surface. The tip was constructed as a 118° cone with a 40 nm radius at the apex. From the comparison with the actual tip, the reconstruction fits the tip well only on the 5 nm apex but is not coincident with the parts below. Of course, this phenomenon may be limited by the maximum height of the sample. Another example is a tip with ∼38 nm-wide base that is simulated by Flater in 2013 [38] with a maximum height of 254 nm. We can clearly see the estimated tip only fits well around the tip apex, but matches poorly the actual tip geometry far from the tip apex even if the maximum peak-to-valley height of the sample is 290 nm. According to their analysis, the reason is that the samples are not optimized to give tip reconstructions that most accurately reproduce the actual tip shape. The reconstructed tip is far from satisfactory even if the features of the samples are smaller than that of the tip. Actually, in previously published papers, the descriptions of suitable tip characterizer are very ambiguous. The authors just use some specific samples to reconstruct some specific tips without regularity conclusions.

In order to explore the errors of BTR caused by sample features, here we carried out a series of simulation experiments based on the conical model. It is generally believed that the reconstructed tip shape fits very well to the real tip shape by using samples with surface structures sharper than the applied tip. However, the simulation results verified that this view is not entirely correct. The prerequisite of the tip characterizer is that its cone angle must be smaller than that of the tip. Then the smaller the radius of curvature of the tip characterizer is, the closer the reconstructed tip is to the actual tip. Through further analysis, we simulate a sharp-edged cylinder structure whose equivalent cone angle is smaller than that of the tip. The results show that this kind of structures is also a suitable tip characterizer.

## 2. Methodology

In this part, we mainly explain the blind reconstruction methods and illustrate some significant equations. For detailed information, please refer to [32] and for detailed principles on how to select tip reconstruction parameters, like tip matrix size and threshold values, please refer to [38].

### 2.1. Image Production

The imaging process can be described as incessantly adjusting the height of the tip until a measured feedback quantity reaching the preset value when it scans the sample point-by-point, line by line. The position of the tip apex will mark the image height at that point. Feedback quantities are selected depending on their sensitivity to the proximity of the tip to the sample surface. The above process approximates to lower the tip until it just touches or nearly touches the sample surface. On the basis of the approximation, the mathematical description of the imaging process is described by Equation (1).


(1) $$i\left(x\prime ,y\prime \right)=-\underset{\left(x,y\right)}{\min}\left[t\left(x-x\prime ,y-y\prime \right)-s\left(x,y\right)\right]$$

Here, *t*(*x* − *x*′, *y* − *y*′) is a function describing the translated tip apex from the point (*x*, *y*). *s*(*x*, *y*) is the top surface of the sample. *i*(*x*′, *y*′) is the image surface.

Then through a few mathematical derivations [32], the imaging equation can be expressed in terms of mathematical morphology as follows.


(2) I=S⊕P

Here, *I* and *S* are the sets of function *i* and *s* respectively describing the top surface of image and specimen. *P* are the sets of function *p* which is a tip reflection through the origin of a coordinate system. ⊕ is the mathematical operation of dilation. Based on the Equation (2), we can use *S* and *P* as inputs to obtain image *I*.

### 2.2. Tip Reconstruction

The BTR method is an iterative process expressed by a mathematical morphology operation as in Equation (3).


(3) $$\begin{array}{ll} P_{i+1}=\underset{x\in I}{\cap }\left[\left(I-x\right)⊕P_{i}^{'}\left(x\right)\right]\cap P_{i}, & P_{i}^{'}(x)=P_{i}\cap \left(x-I\right) \end{array}$$

*P_i_* is the iteration result of the reflection tip. $P_{i}^{'}$excludes the forbidden situation that the tip apex penetrate the sample. *I* is an image surface set.

Since the tip-sample touching point is unknown, the BTR algorithm gets the contact coordinates by comparing every allowable touching point for a given image and tip coordinate. As Villarubia verified [32], each iteration of Equation (3) will produce a result smaller than or equal to the preceding one. Until the convergence limit, *i.e.* no modification of the tip, the best estimated tip will be obtained by blind reconstruction procedure. For a more detailed implementation of Equation (3), please refer to [32].

**Figure 1.**
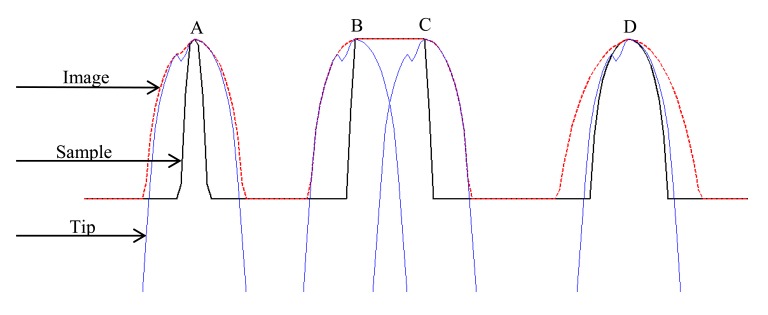
Comparison of the sample, image and the inverted tips.

From the imaging process, it is obvious that the sample is a subset of the image, or equivalently, the top surface of the image forms an upper bound on the top of the sample as shown in Figure 1. The image is formed by dilation operation between sample and tip. Switching the two factors will provide the same result according to the reflexive property of dilation. Consequently, features on the image can be regarded as broadened images of the inverted tip that has been imaged by sample features. Owing to the fact that tips are generally much smaller than the samples in size, we can safely assume that the interaction parts between tip and sample at each scanning point is very limited compared with the whole sample. In this way, it is feasible to regard the sufficiently separated subsets of the image as independent parts, each of which respectively places an upper bound over the tip just like the four features named A, B, C and D in Figure 1. Therefore it would be better to extract more effective tip geometry if the useful features of the sample separate far enough.

In order to obtain accurate tip shape, the image surface shape must coincide with or be very close to the inverted tip at each local maximum point of image as shown in A, B, and C features. What need to be emphasized is that sometimes if the sample feature is inappropriate, the reconstructed tip will be much wider than the actual tip. As shown in D, the size of the image at this local maximum point almost doubles with respect to the size of the sample in the horizontal direction. This will lead to the result that the reconstructed tip is almost twice wider than the size of the actual tip, since BTR algorithm completely depends on images to reconstruct tip shape, and in this case, the reconstructed tip shape will be equal to the image of the feature. This is why we need to choose suitable sample to obtain more accurate 3D tip shape.

### 2.3. Surface Reconstruction

With a given image *I* and an estimated tip shape *P*, we can reconstruct the sample surface by the mathematical erosion operation as follows.


(4) $$S_{r}=I\Theta P$$

First of all, if the estimated tip shape is the same as actual tip shape, *S_r_* is not only an upper bound on the actual sample surface but also the best possible reconstruction. That is to say no upper bound smaller than *S_r_* is acceptable and *S_r_* is the least upper bound consistent with the image [32]. Why it is simply the upper bound rather than actual sample surface even though the actual tip shape is applied. The reason is that sometimes the tip is too large to penetrate regions like undercuts, narrow ditches or the base of steep walls. In summary, we can conclude that the erosion method can reconstruct all the regions of the sample surface that are touched by the tip. This in turn provides an interpretation why this method can obtain the best possible reconstruction. On the other hand, if the reconstructed tip shape is just an outer bound of the actual tip, some parts of the sample surface reconstructed by such a tip through erosion algorithm may be below the corresponding parts of the actual sample surface while the others are upper bound. In this case, the reconstructed sample surface is neither the upper nor lower bound of the real sample surface, which makes it more difficult for us to estimate the real sample surface. This phenomenon indicates how necessary it is to select the suitable sample.

In addition, based on the commutative law, the erosion method provides another great service shown in Equation (5).


(5) $$P_{r}=I\Theta S$$

It means we can use erosion to reconstruct the tip shape if the sample surface geometry *S* is known while the tip shape is not. Strongly resembling the foregoing discussions, *P_r_* is only equal to those segments of *P* that have been in contacted with the sample surface but an upper bound elsewhere. Putting another way, Equation (5) can return to all sample-tip touching points of the real tip which is helpful to examine if the selected sample contains suitable structures.

## 3. Simulation Description and Discussion

### 3.1. Simulation Description

**Figure 2.**
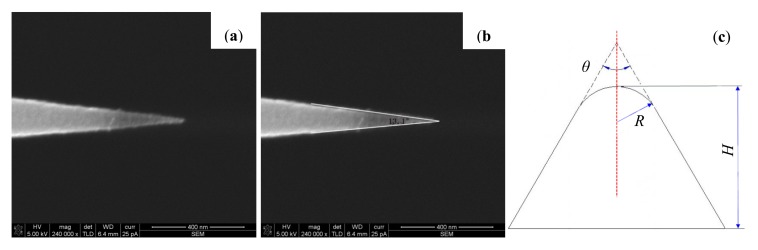
(**a**) A SEM image of measured tip NO.5; (**b**) A duplicate of image (a) with the cone angle lines; (**c**) A center cross-section of the supposed conical tip model.

As for the high resolution of CSG01 series from NT-MDT company, the parameters of the probes on the specification of CSG01 series is as follows: tip height varies from 10 μm to 15 μm, tip cone angle is no more than 20° and the tip radius of curvature is small than 10 nm. In order to know the accurate parameters of this kind of probes, nine unused probes have been imaged by SEM. Figure 2a shows a SEM photo of tip NO.5. The cone angle θ of the tip is measured for a height H of ∼350 nm (Figure 2c).The other parameters are listed in the following Table 1. The averages of the tip radius of curvature and cone angle are ∼9.2 nm and 16.1°, respectively, which is in accord with data from the manufacturer. According to the actual probe shape, we suppose the tip is a circular cone tangent to a sphere on the top. The cross sections through the apex of the supposed tip model are illustrated in Figure 2b.

In order to explore the sources of errors of BTR, a representative set of simulation tests are implemented in this section. The simulation platforms we are using here are MATLAB R2012a and Microsoft Visual Studio 2010. These tips and samples are simulated based on 3D geometrical model on MATLAB. The images are generated by the dilation algorithm in Equation (2) and the tips are reconstructed by the iterative algorithm in Equation (3) on Microsoft Visual Studio. The source codes of the above two algorithms were provided in C code by Villarrubia [32]. The samples are simulated with analogous structure as the upper mentioned tip but with different parameters. Since it is not possible for any technique to reconstruct tip parts that are not touched by sample features, the cone angle of the samples must be smaller than that of the tip to touch it as much as possible. First, three sets of experiments with tip cone angle of 16° and sample cone angle of 10°, 15° and 20° respectively were done to verify this inference. Then based on the parameters of the aforementioned actual tips and the wear effects which make the tip flatten during the scanning process, we suppose the tip cone angles are equal to the above average value of 16° and the tip radii of curvature are 20 nm, 50 nm and 80 nm respectively. Hence three sets of experiments were carried out to investigate the effect of tip radius on tip reconstruction. Finally, another three sets of experiments with tip cone angle of 16°, 32° and 64° were performed to investigate the influence of the tip cone angle on the tip reconstruction. All sets of experiments were carried out with samples of different radius of curvature. The detailed parameters and errors are illustrated in Table 2. The sample matrix sizes are 100 × 100 and the maximum height are 100 nm. The matrix sizes of tips range from 35 × 35 to 49 × 49 based on the actual tips sizes. The heights of the tips are 120 nm.

The estimated tip shape was obtained from the ideal noise-free image. In AFM imaging, we can change the lateral resolution by setting different scan size and pixel numbers. Such as, when images are collected on the same area and at the same scan size of 5 μm, but with pixel numbers of 512 × 512, 256 × 256, the lateral resolution is up to ∼9.76 nm/pixel and ∼19.53 nm/pixel respectively. Resembling the AFM lateral resolution, via 2D interpolation procedure (The essential part of “2D interpolation” procedure is the function of “interp2” in MATLAB), we present the samples and tips matrix sizes of the same resolution of 4 nm/pixel, which is smaller than the above mentioned lateral resolution. Just like we can choose different pixel numbers in the AFM imaging, the essence of this procedure is to reduce the pixel numbers of the tips and samples, so we can reduce the matrix sizes of samples and tips without changing their shapes. (Note that when using 2D matrix to show 3D morphology in MATLAB, the matrix sizes are the pixel numbers). The decrease of matrix sizes is of great help to reduce the time of tip reconstruction, and the reconstruction times are all shorter than 180 s in the simulated experiments. For example, for the image with 100 × 100 points and the tip with 41 × 41 points, the reconstruction time is around 120 s. Apparently, time decrease is at the cost of lower resolution. In order to balance the relationship between the resolution and time, we can choose samples with specific features instead of arbitrary structure to reduce the scan size. Then with the same lateral resolution, the pixel numbers will decrease. Finally, it will cause the decreasing of the reconstructed time.

**Figure 3.**
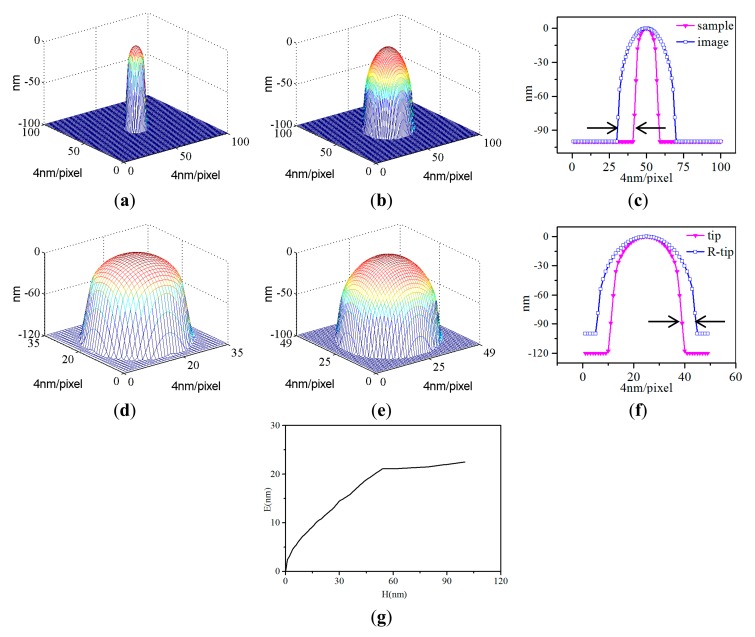
An example of simulated experiments using the Blind Tip Reconstruction (BTR) method. (**a**) A simulated 3D sample surface; (**b**) An image obtained by dilating sample surface with the simulated tip; (**c**) Comparison of cross sections through the apex of the sample and image; (**d**) A simulated 3D tip; (**e**) A reconstructed 3D tip produced by using the created image in BTR algorithm; (**f**) Comparison of cross sections through the apex of the tip and reconstructed tip; (**g**) The deviations between the actual tip and the reconstructed tip. H means the tip height measured from the tip apex. E means the deviations indicated by the black arrows in the same height in Figure 3f.

Here we illustrate one experiment ((*R*_t_, *C*_t_, *C*_s_) is (50 nm, 16°, 15°), *R*_s_ is 25 nm) in Figure 3 to make the whole simulation process clearly.

*R*_s_ means the radius of the sample. *R*_t_ means the radius of the tip. *C*_t_ means the cone angle of the tip. Cs means the cone angle of the sample. In comparison with the simulated 3D sample in Figure 3a, the image obtained by dilating sample surface with the simulated tip in Figure 3b is obviously dilated by the tip shape. Figure 3c shows the comparison of cross sections through the apex of the sample and image. The black arrows in Figure 3c indicate the error between the sample surface and image at the height of ∼90 nm below the apex, which is about 42 nm. The size of the image almost doubles with respect to the size of the sample. Therefore, it is crucial to extract the tip shape and reconstruct the sample. In Figure 3d, we can see the height of the tip is 20 nm higher than that of the sample of 100 nm. In addition, the height of the reconstructed tip which is also 100 nm as seen in Figure 3e is just equal to that of the sample, which means the height of the reconstructed tip is determined by the height of the tip characterizer. Whatever the required height of the reconstructed tip, a tip characterizer of the same or larger height should be used. Therefore, here we concentrate on the errors in width, while ignoring the errors along the height.

Figure 3f shows the deviations between the actual tip and the reconstructed tip. The black arrows in Figure 3f, where the two curves tend to be parallel below ∼60 nm, indicate the errors of BTR presented in Table 2. As we can see, the errors are defined as the stable difference in half widths between the actual tip and the reconstructed tip. Note that the deviations between the tips are different at different places. This definition for error does not take into account the entire geometry of the tip. However, in this paper, we simply use error to evaluate the quality of the reconstructed results. In addition, in Figure 3g, we can see the errors increase linearly with the tip heights and will tend to be stable when the tip heights are high enough, so the stable error can achieve this objective. Furthermore, this phenomenon appeared in other experiments in Table 2, since the heights of the samples were all made high enough to provide reconstructed tips of enough height. Hence, here we choose the stable errors as the error of BTR.

**Figure 4.**
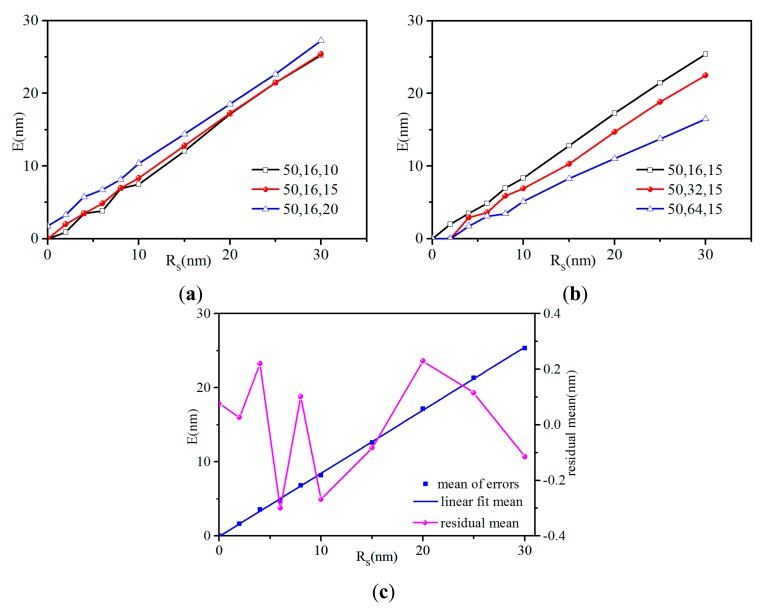
The errors between the reconstructed tip and the actual tip obtained by various samples and tips. (**a**) The comparison of the errors (*E* on the Y axis) between (50, 16, 20), (50, 16, 15) and (50, 16, 10); (**b**) The comparison of the errors between (50, 16, 15), (50, 32, 15) and (50, 64, 15); (**c**) The mean of the errors between (80, 16, 15), (50, 16, 15) and (20, 16, 15) and associated linear fit. The residual mean is the deviation between the mean values calculated for each *R*_s_ and their fitted counterparts.

From the study of the influence of the radius of curvature and the cone angle in the frame of the conical model, three conclusions can be drawn. First, in Table 2, the error of (50, 16, 20) is 1.76 nm even if the radius of curvature of the tip characterizer is 0 nm, while the others whose cone angles are smaller than that of the tips are 0 nm. Furthermore, as shown in Figure 4a, the errors of (50, 16, 20) are larger than that of the two others and the curve is roughly parallel to the other two curves. Hence, to reconstruct a tip shape completely consistent with the actual one, the prerequisite is that the cone angle of the tip characterizer must be smaller than that of the tip. The curves of (50, 16, 15) and (50, 16, 10) in Figure 4a roughly coincide with each other, which shows that the errors are insensitive to the cone angle of tip characterizer when it is smaller than that of the actual tip. Second, for tips with the same radius of curvature and different cone angle, the comparison of (50, 16, 15), (50, 32, 15) and (50, 64, 15) in Figure 4b shows that the errors decrease when the tip cone angle increases if the cone angle of the tip characterizer is smaller than that of the actual tip. The errors are sensitive to the cone angle of the tip: the larger the cone angle of the tip is, the more accurate the reconstructed tip is. Third, even if the radius of curvature and the cone angle of the tip characterizer are smaller than their tip analogs ((*R*_t_, *C*_t_, *C*_s_) is (80 nm, 16°, 15°), *R*_s_ is 30 nm)), the calculated error remains unexpectedly large (24.95 nm). For tips with the same cone angle and different radius of curvature, the errors associated with (80, 16, 15), (50, 16, 15) and (20, 16, 15) tips are almost the same as shown in Table 2. They increase linearly with the radius of curvature of tip characterizer as depicted in Figure 4c. Whatever the size of tip radius of curvature is, to reconstruct an accurate tip shape, the radius of curvature of tip characterizer should be as small as possible. When it is smaller than 5 nm, the errors are ∼4 nm.

### 3.2. Local Error Model of BTR

**Figure 5.**
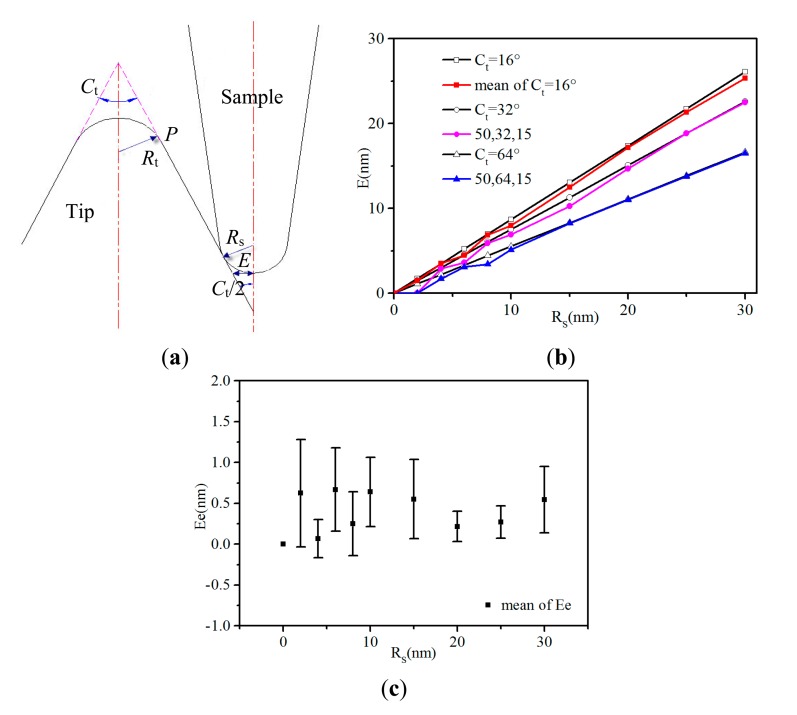
(**a**) The local error model of BTR allowing errors of the BTR method to be calculated; (**b**) The comparison of the errors from the Equation (6) (the black straight lines with open symbols) and the simulated experiments (the color curves with solid symbols). *C_t_* = 16°, *C_t_* = 32° and *C_t_* = 64° are the errors calculated from the Equation (6) when tip cone angles are 16°, 32° and 64° respectively. Mean of *C_t_* = 16° are the average errors of (50, 16, 15), (50, 16, 10), (80, 16, 15) and (20, 16, 15), since they have the same cone angle of 16°. The two other curves are the errors of (50, 32, 15) and (50, 64, 15), whose tip cone angles are 32° and 64° respectively; (**c**) The average deviations between the errors from the Equation (6) and the errors derived from the simulated experiments of (50, 16, 15) (50, 16, 10) (80, 16, 15) (20, 16, 15) (50, 32, 15) and (50, 64, 15) at different tip cone angles. Error bars represent the corresponding standard deviations.

On the basis of the previous simulation results, we present now the local error model of BTR calculating BTR errors (Figure 5a). This model is equivalent to a reverse AFM imaging process where the tip would be scanned by the sample. In the frame of this model, the cone angle of the sample is assumed to be smaller than that of the actual tip. The error (the distance *E* in Figure 5a) of the BTR method is described by Equation (6).

(6) $$E=R_{\text{s}}\left(1-\sin\left(C_{\text{t}}/2\right)\right)/\cos\left(C_{t}/2\right)$$

here, *R_s_* is the radius of curvature of the sample and *C_t_* is the cone angle of the tip. The error *E* corresponds to the stable error mentioned in Section 3.1. It decreases as a function of the tip cone angle and increases linearly with the sample radius of curvature just as the above stable error derived from the simulated experiments. Since it only relates to the radius of curvature of the sample and the cone angle of the tip, the error *E* will stay the same until the contact point just moving up to the tangential point *P* of the tip in Figure 5a. The tangential point is a critical point. Above this point, the errors do not level off when changing tip height. The tip height at this point can be described by the following Equation (7).

(7) $$H=\left(R_{t}+R_{\text{s}}\right)\left(1-\sin\left(C_{\text{t}}/2\right)\right)$$

here, *H* is the tip height measured from the tip apex at the critical point. When it gets higher, the error will stay the same. *R_s_* is the radius of curvature of the sample and *C_t_* is the cone angle of the tip.

**Figure 6.**
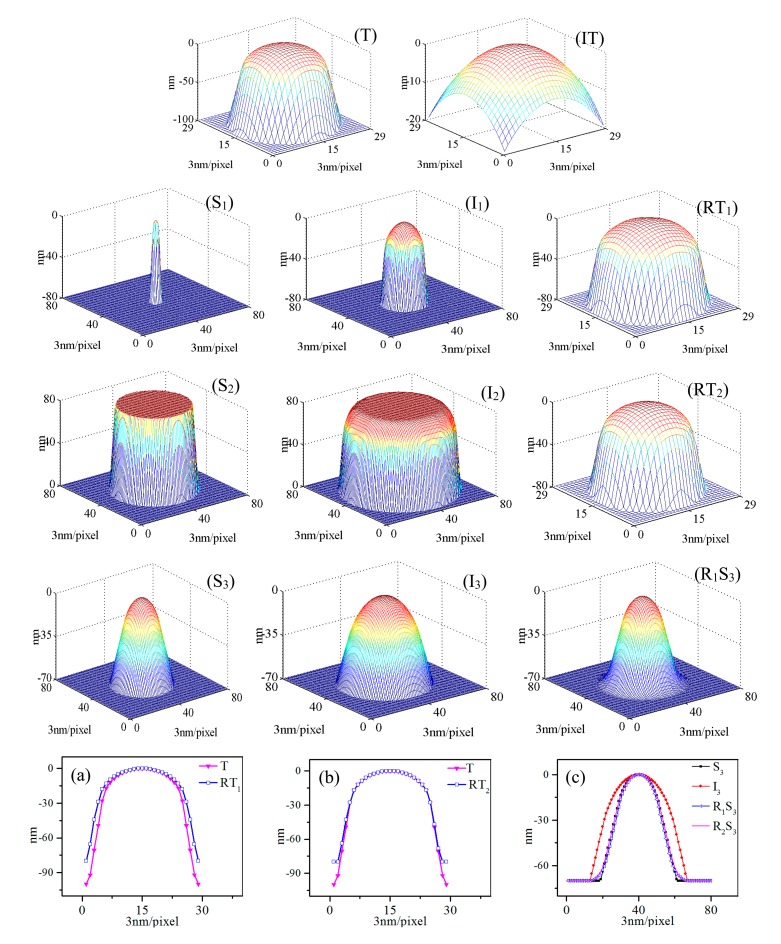
(**T**) A simulated 3D tip. (**IT**) an initial 3D tip for S_2_. (**S_1_**,**S_2_**) are simulated suitable structures for BTR. (**S_3_**) is the sample needed to be reconstructed. (**I_1_**,**I_2_**,**I_3_**) are the images obtained by dilating sample surface with the simulated tip. (**RT_1_**) and (**RT_2_**) are the reconstructed tips produced by using the created image I_1_ and I_2_ in BTR algorithm. (**R_1_S_3_**) is the reconstructed sample surface produced by using the reconstructed tip (RT_1_) in erosion algorithm. (**a**) The comparisons of cross sections through the apex of the actual tip (T) and the tip reconstructed from S_1_ (RT_1_); (**b**) The comparisons of cross sections through the apex of the actual tip (T) and the tip reconstructed from S_2_ (RT_2_); (**c**) The comparisons of cross sections through the apex of the actual surface of S_3_, image (I_3_), reconstructed image using RT_1_ (R_1_S_3_) and reconstructed image using RT_2_ (R_2_S_3_).

As seen in Figure 5b, the errors calculated using Equation (6) are in good agreement with those derived from the simulated experiments. They are sensitive to the radius of curvature of the samples and the cone angle of the tip but insensitive to the radius of curvature of the tip and the cone angle of the samples when the cone angle of the sample is smaller than that of the tip. The maximum deviation is 1.22 nm. Figure 6c shows the corresponding average deviations and standard deviations (*E*_e_ on the Y axis) of Figure 6b at different radius of curvature of the samples. As seen in Figure 6c, the deviations vary with the radius of curvature of the samples. They disperse greatly and there are no rules between them. When the radius of curvature of the samples is 2 nm, the deviation can be expressed as 0.62 ± 0.66 nm. The overall deviation can be expressed as 0.43 ± 0.83 nm. Combined with the above mentioned lateral resolution of AFM, which can be up to ∼3.9 nm/pixel when the scan size is 1500 nm and pixel numbers are 512 × 512. In addition, increasing the scan size or decreasing the pixel numbers, the lateral resolution will become larger. Compared with the lateral resolution used in the above simulated experiments (4 nm/pixel), it is valid to ignore the deviations between the errors derived from the Equation (6) and the simulated experiments. Hence, the local error model of BTR is indeed in quantitative agreement with the simulation results.

Since the errors calculated from Equation (6) closely match those derived from the simulated experiments, we can consider the reconstructed tip as a broadened image of the tip. In order to obtain an accurate tip shape, the image must coincide with or be very close to the inverted tip shape. Hence, the cone angle of the tip characterizer must be smaller than that of the tip to make most parts of the tip touch by the tip characterizer end. Furthermore, the smaller the radius of curvature of the tip characterizer is, the closer the geometries of the image and the tip are. When it is infinitely close to 0 nm, the geometries of the image and the tip will be the same. In this situation, BTR method will reconstruct tip exactly as the actual tip. The tip characterizer end can be extended to the local maximum point of sample and the radius of curvature can be extended to the roundings of the local maximum point. These conclusions will provide great help for users to choose suitable tip characterizer so as to reconstruct accurate 3D tip shape and to get the actual sample surface.

### 3.3. Suitable Structure

On the basis of the previous analysis, in Figure 6, we propose two suitable structures for a better reconstruction of BTR method. A 3D tip with cone angle of 16° and radius of curvature of 30 nm was simulated as shown in Figure 6T. Figure 6S_1_ is a conical structure S_1_ with small radius of curvature. The cone angle and radius of curvature of S_1_ are 10° and 5 nm. Figure 6S_2_ is a cylindrical structure S_2_. The corresponding cone angle of S_2_ is 15°. According to the previous conclusions in Section 3.2, the cone angles of S_1_ and S_2_ are all smaller than that of the tip (S_1_ and S_2_ are sharper than the tip). Figure 6S_3_ is a sample S_3_ with cone angle of 60° and radius of curvature of 40 nm. In Figure 6I_1_–I_3_, we can clearly see the images are distortions and expansions of the original samples. As shown in Figure 6RT_1_, the tip reconstructed from S_1_ is a very close outer bound of the actual tip. The error is ∼3.69 nm as can be seen in Figure 6a. The reconstructed S_3_ is over eroded by the reconstructed tip as seen in Figure 6c. However, the error is ∼2.42 nm much smaller than the image error which is ∼18.74 nm as shown in Figure 6c. In Figure 6RT_2_, the S_2_ structure provided a 3D tip shape which is almost the same as the actual one. The error between the actual tip (T) and the tip reconstructed from S_2_ (RT_2_) is only ∼0.95 nm as seen in Figure 6b. In addition, Figure 6c shows that the reconstructed image using RT_2_ is very similar to the actual sample surface. The error between them is nearly 0 nm. However, note that the initial tip shape leading to this tiny error must be a specific curve structure as represented in Figure 6IT.

The simulation results verified the above conclusions, especially the conclusion derived from Section 3.2 that the radius of curvature can be extended to the roundings of the local maximum point. From the comparison of Figure 6a,b, the cylindrical structure S_2_ provided better reconstruction than that of the conical structure S_1_. For structure S_2_, there are no roundings of the local maximum point which means the radius of curvature is ∼0 nm. That is why the S_2_ structure provided a 3D tip shape which is almost the same as the actual one.

## 4. Conclusions

In order to get a better reconstruction of 3D tip shape and sample surface, we first need to understand that the reconstructed tip height is determined by the height of the tip characterizer. One should choose it higher than the height of samples that needed to be reconstructed. Furthermore, to make features on AFM images close to the geometry of the tip, the cone angle of the tip characterizer must be smaller than that of the tip. When it meets the above conditions, the reconstructed tip is insensitive to the cone angle of the tip characterizer based on the previous simulation experiments and analysis. The BTR errors are insensitive to the radius of curvature of the tip but sensitive to the tip cone angle. They decrease as a function of the tip cone angle. With the same tip characterizer, for tips with large cone angles, the errors of BTR are smaller than the tips with small cone angles.

The reconstructed tip is very sensitive to the radius of curvature of the sample. The smaller it is, the better the result is. In an ideal situation, the sample is a circular cone with one peak point. Unfortunately, it is hard to find such samples in reality, but, when the radius of the tip characterizer is smaller than 5 nm, the error will be around 4 nm. The reconstructed sample surface will be much closer to actual sample surface than the image. For example, the ‘Tipcheck’ sample (RS-12M) from Bruker Corporation containing many very sharp grain features of radii smaller than 5 nm combined with a cliffy and higher grating whose equivalent cone angle is smaller than that of the tip, will be ideal for determining the tip shape in the BTR method. The cylindrical structure, whose equivalent cone angle is smaller than that of the tip, is also a good tip characterizer for tip reconstruction, such as, ultra-precision turning of micro-optical components which contain sharp edges like Fresnel reflectors. However, no absolute prismatic structures like the ideal cylindrical structures in reality. There will always be roundings around the local maximum points of the cylindrical structures. The roundings of the local maximum points are similar to the radius of curvature of conical structures. The bigger the rounding radius, the larger the error is.

## Figures and Tables

**Table 1.** The parameters of nine unused probes (CSG01) obtained from SEM images.

**Probe Number**	**1**	**2**	**3**	**4**	**5**	**6**	**7**	**8**	**9**	**Average**
Tip radius of curvature (nm)	9.4	9.35	10.0	9.15	9.35	10.0	8.85	7.8	8.9	9.2
Tip cone angle *θ* (°)	19.2	17.4	14.0	15.8	13.1	12.8	16.4	16.8	19.5	16.1

**Table 2.** The errors of the BTR of different tips and samples. For example, on row of (80, 16, 15), it shows how the errors change with the radius of curvature of samples when the radius of curvature of tip is 80nm, the cone angle of tip is 16° and the cone angle of sample is 15°.

***R*_s_(nm)**	**30**	**25**	**20**	**15**	**10**	**8**	**6**	**4**	**2**	**0**

**(*R*_t_,*C*_t_,*C*_s_) (nm,°,°)**										
(80,16,15)	24.95	21.20	16.89	12.05	7.62	6.60	4.22	3.65	1.35	0
(50,16,15)	25.40	21.48	17.29	12.82	8.33	6.95	4.86	3.48	2.00	0
(20,16,15)	25.71	21.30	17.37	12.97	8.56	6.96	5.12	3.52	1.61	0
(50,32,15)	22.49	18.84	14.70	10.28	6.91	5.90	3.61	2.93	0	0
(50,64,15)	16.50	13.75	11.01	8.26	5.12	3.43	3.09	1.71	0	0
(50,16,10)	25.24	21.43	17.16	12.08	7.47	6.92	3.82	3.46	0.88	0
(50,16,20)	27.26	22.63	18.53	14.41	10.35	8.17	6.73	5.74	3.25	1.76
